# Utility of the Serum Protein Electrophoresis in the Opportunistic Screening for the Deficiency of Alpha-1 Antitrypsin

**DOI:** 10.3390/diagnostics13172778

**Published:** 2023-08-28

**Authors:** Beatriz Fernández-Gomez, Sebastian Menao-Guillén, Ayla Fernandez Gonzalez, Maria Arruebo Muñio, Monica Ramos Alvarez, Mercedes Inda Landaluce, Maria Angeles Castillo Arce, Miguel Ángel Torralba-Cabeza

**Affiliations:** 1 Faculty of Medicine, University of Zaragoza, 50009 Zaragoza, Spain; beatrizfernandezgomezc@gmail.com; 2 Department of Biochemistry, “Lozano Blesa” University Hospital, 50009 Zaragoza, Spain; smenao@hotmail.com (S.M.-G.); aylafg94@gmail.com (A.F.G.); mam.arruebo@gmail.com (M.A.M.); monramalv@yahoo.es (M.R.A.); mminda@salud.aragon.es (M.I.L.); macastilloarce@gmail.com (M.A.C.A.); 3 Instituto de Investigación Sanitaria de Aragon, 13th San Juan Bosco Avenue, 50009 Zaragoza, Spain; 4 Unit or Rare Disorders, Department of Internal Medicine, “Lozano Blesa” University Hospital, 15th San Juan Bosco Avenue, 50009 Zaragoza, Spain; 5 Working Group on Minority Diseases of the Spanish Society of Internal Medicine (GTEM-SEMI), 50009 Zaragoza, Spain

**Keywords:** alpha-1 antitrypsin, protein electrophoresis, *SERPINA1*, screening, genetic counseling

## Abstract

Background: A deficiency in alpha-1 antitrypsin (AAT1) is a rare disorder that represents a significant health threat and early diagnostic priority issue. We investigated the usefulness of the serum protein electrophoresis (SPE) as an opportunistic screening tool for AAT1 deficiency. Methods: For 6 months, all SPE carried out for any reasons were evaluated in our center. In those with less than 3% of alpha-1 globulins, AAT1 concentrations were studied. The *SERPINA1* gene was subsequently sequenced in those patients displaying concentrations below 100 mg/dL. Results: Out of the total, 14 patients (0.3%) were identified with low AAT1 concentrations, with 11 of them agreeing to enter the study. Of those, mutations in the *SERPINA1* gene were discovered in 10 patients (91%). Heterozygous mutations were detected in seven patients; three had the c.1096G>A mutation (p.Glu366Lys; Pi*Z), two had the c.863A>T mutation (p.Glu288Val; Pi*S), one had the c.221_223delTCT mutation (p.Phe76del; Pi*Malton), and the last one had the c.1066G>A (p.Ala356Thr) mutation, which was not previously described. Finally, one patient had the c.863A>T mutation in homozygosis, whereas two double heterozygous patients c.863A>T/c.1096G>A were detected. Conclusions: An altered result in the concentration of AAT1 anticipates a mutation in the *SERPINA1* gene in a manner close to 91%. The relationship between a decrease in the alpha-1 globulin band of the SPE and an alteration in the AAT1 concentration is direct in basal states of health. The SPE is presented as a highly sensitive test for opportunistic screening of AAT1 deficiency.

## 1. Introduction

Alpha-1 antitrypsin (AAT1) deficiency (MIM 613490) is an autosomal co-dominant disorder classified as a rare disease. AAT1 is an inhibitor of serine proteases, especially neutrophilic elastase. When an imbalance occurs between protease and antiprotease levels, pulmonary pathology can develop early [1,2]. The most common manifestation is emphysema, which becomes evident by the third or fourth decade. A less common manifestation of this entity is cirrhosis or liver failure, which occurs in children and adults. The liver pathology is due to a toxic gain of function related to intrahepatic polymerization of some of the variant proteins [3].

AAT1 is the most abundant serum antiproteinase [4]. It is an acute phase glycoprotein formed by 394 amino acids, arranged in three beta sheets and a mobile reactive zone. AAT1 is produced in hepatocytes and is encoded by the *SERPINA1* (Pi) gene (OMIM 107400) that is located on chromosome 14q32.1. The plasma concentration should range between 100 and 200 mg per deciliter and the liver produces approximately 34mg of AAT1 per kilogram of body weight per day, although this amount may be increased in response to inflammatory or tumor type processes. The AAT1 presented in serum is 40% of the total, with the remaining 60% staying in the extracellular space. The AAT1 deficiency is a consequence of mutations in the gene that encodes it. To date, more than 70 variants have been described in the *SERPINA1* gene, of which, at least 30 have pathological consequences [5,6,7]. The most common genetic mutations causing decreased release of AAT1 to serum are called Pi*Z (c.1096G>A;p.Glu366Lys) and Pi*S (c.863A>T;p.Glu288Val). In addition, Pi*ZZ and Pi*SZ are the most frequent severe deficient phenotypes.

Common methods for diagnosing AAT1 deficiency include quantification of serum, phenotyping, and genotyping of AAT1 [8]. In the event of the diagnosis of a congenital deficiency, it is assumed that values below 35% of normality indicate the possibility of Pi*ZZ. In this determination, it is necessary to consider that the AAT1 is an acute phase reactant and, therefore, it can be elevated with non-specific inflammatory or infectious activity. High artifact values have also been described in pregnancy and the use of oral contraceptives [6]. In addition, only with quantification, although the measurement is performed very accurately, patients with heterozygous mutations that have AAT1 levels at the low limit of normality can be overlooked [9]. In general, homozygous people have low serum AAT1 levels, and after that discovery, the AAT1 phenotype for confirming AAT1 deficiency is studied [10]. The molecular analysis of the AAT1 gene is the reference method to identify the less frequent allelic variants related to the deficiency, the null variants, or for the characterization of new variants [11,12,13].

There is no doubt of the importance of diagnosing patients with AAT1 deficiency, not only to start treatment, if indicated, but also to study the first-degree relatives and offer them genetic counseling to establish preventive measures and complete care based on their diagnosis. It is estimated that in Spain, the AAT1 deficiency affects 1 in 2500 people, but according to the Spanish Registry of Patients with alpha-1 Antitrypsin Deficiency, only 500 people have been diagnosed with the Pi*ZZ mutation [14].

The serum protein electrophoresis (SPE) is a laboratory technique that allows proteins to be separated based on their displacement when they are subjected to an electric field and classifies circulating proteins as albumin and globulins. A normal SPE can be considered with the following values: Albumin 53–69%; 35–46 g/L, alpha-1 globulin: 3–4%; 1–3 g/L, Alpha-2 globulin: 6–11%; 4–7.5 g/L, Beta globulin: 8–13%; 5–9 g/L, and Gamma globulin: 12–19%; 8–12 g/L. Since alpha-1 antitrypsin is the most abundant globulin in the alpha-1 group, if a patient has a lower AAT1 concentration than average, it can be represented by a band in the diminished alpha-1. This determination is, therefore, qualitative and may indicate normal, intermediate, or low enzyme values. The SPE is a test that is requested by various medical specialties, mainly in the search of monoclonal bands, and occasionally low values of the alpha region are noted. The objective of this study was to assess a detection strategy for patients with mutations in *SERPINA1* gene based on the incidental finding of low values of the alpha-1 region of the SPE given that it is an underdiagnosed and potentially treatable disease.

## 2. Material and Methods

This study was performed on all the SPE (capillary serum electrophoresis in free solution) carried out for any reason from September 2019 to February 2020 in the Laboratory of Biochemistry of the “Lozano Blesa” University Hospital of Zaragoza, Spain. We selected a group of participants older than 18 years, in which the quantification of the alpha-1 globulin band was <3%. The conditions of the SPE were: on a volume of 30 uL of the sample, an average voltage of 20,000 volts were applied to the final extremes of the capillary for 10–30 s, achieving an electric field that separates the proteins through the buffer vials, being essential to maintain the temperature to avoid thermal gradients that suppose distortions. Column separation was monitored using ultraviolet-visible (UV-Vis) light at a wavelength of 214 nm.

Subsequently, the concentration of AAT1 was determined in them, and those in which a concentration lower than 100 mg/dL were detected as participants under study.

After the identification of the possible cases, they were contacted by telephone and those who agreed to participate in the study had a blood sample taken, in addition to conducting a clinical interview recording the family history. The blood collection tubes were labelled, registering the day and time of extraction, and were afterwards processed and stored in the appropriate conditions. This study was carried out following the principles of the Helsinki Declaration, being approved by the Ethics and Research Committee of Aragón (CEICA) Ethics and Research Committee of Aragón (CEICA) (Approval Code: CI PI20/219; Approval Date: v3.14/05/2020).

To determine the serum AAT1 concentration, an immunonephelometry method was used in BN* II and BN ProSpec^®^ systems. The genetic study of the coding exons and adjacent intronic regions of the *SERPINA1* gene (ENST00000448921.5) was carried out with the following primers (Table 1). The GenBank accession number (Gencode Gene) was ENSG00000197249.14.

The amplification conditions were as follows: 5 min of preamplification at 95 °C, 35 cycles of amplification (30 s at 95 °C, 30 s at 60 °C and 30 s at 72 °C), and, finally, a post-amplification cycle (10 min at 72 °C). After subsequent verification of the correct amplification, sequencing was performed by capillary electrophoresis (ABI 3500 XL from Applied Biosystems, Foster City, CA, USA). The DNA polymerase used was VWR Taq DNA Polymerase (Cat. No.: 733-1301).

The databases used to estimate the population frequency of the found variants were the following: (1) gnomAD: https://gnomad.broadinstitute.org/ (Accessed on 18 May 2020); (2) ExAc: http://exac.broadinstitute.org/ (Accessed on 18 May 2020); (3) ESP (NHLBI Exome Sequencing Project): https://evs.gs.washington.edu/EVS/ (Accessed on 18 May 2020), and (4) 1000Genome: https://www.internationalgenome.org/home/ (Accessed on 18 May 2020). The bioinformatics tools used to estimate the splicing site were: (1) Human Splicing Finder: http://www.umd.be/HSF/ (Accessed on 18 May 2020) and (2) Berkeley Drosophila Genome Project: www.fruitfly.org/seq_tools/splice.html (Accessed on 18 May 2020).

Given the characteristics of the study, the statistical model used in this work was descriptive and was carried out using the SPSS 22.0 program, with which the quantitative variables, the mean, standard deviation, minimum, and maximum, were calculated. In the case of qualitative variables, the percentages were calculated.

## 3. Results

A total of 12,800 SPE were studied, of which a percentage of alpha-1 globulins less than 3% was detected in 40 of them (0.31%). After determining the concentration of AAT1, 14 participants (0.11%) showed levels below 100 mg/dL and only 11 of them participated in the study. One of those who did not partake in the study died as a consequence of “idiopathic” liver cirrhosis associating pulmonary emphysema and the other two refused consent.

**Figure 1:**
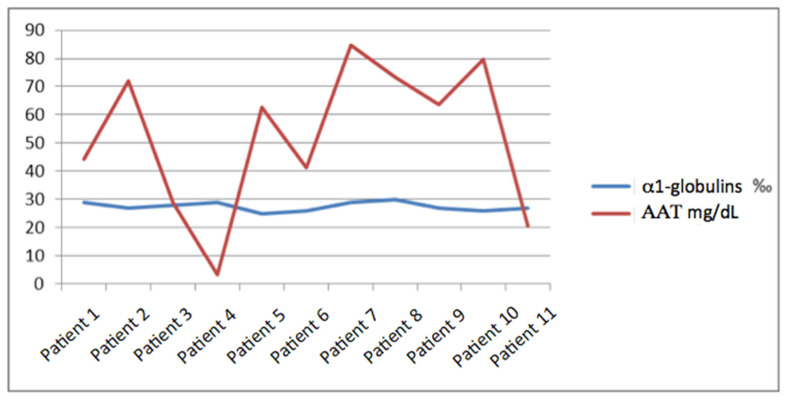
Comparison between the percentage of α1-globulins and the concentration of AAT1.

Regarding the concentration of AAT1, the average was 53.7 mg/dL with a maximum of 84.6 mg/dL and a minimum of <10 mg/dL, with a standard deviation of 26.18. We did not find a relationship between it and the percentage of alpha-1 globulins, and the range in which this percentage moved was narrow (since it did not vary by more than 0.5%) and instead, the margin of AAT1 was much wider with a variation between the maximum and minimum value of more than 70 points (Figure 1).

Out of the eleven patients studied, four were women (36%) and seven were men (64%). Regarding age, they did not present a distribution with a specific pattern, although it is true they were mostly middle aged, with an average of 54 years, a maximum of 90 years, and a minimum of 19, respectively (standard deviation of 22.13). The clinical characteristics of the patients and relatives were studied, where it was remarkably found that two patients suffered infertility problems, two had liver fibrosis, one was affected by psoriasis, and another with celiac sprue. After a family medical record, five of the participants did not have lung or liver diseases between the first degree relatives but some entities were discovered in the family members of the other six (Table 2).

After the demonstration of the enzyme deficiency, the genetic study was carried out, resulting in the finding of mutations in 10 of the 11 patients. With these data, it can be affirmed that in this series, 91% of the altered AAT1 concentrations anticipated the presence of a mutation in any of the exons of the *SERPINA1* gene (Table 3).

Heterozygous mutations were detected in seven patients (NM_001002235.2), three had the c.1096G>A mutation (p.Glu366Lys; Pi*Z), two had the c.863A>T mutation (p.Glu288Val), one had the c.221_223delTCT (p.Phe76del) mutation, and in another, the c.1066G>A mutation that is not currently described. Finally, one patient had the c.863 A>T mutation in homozygosis, whereas two double heterozygous c.863A>T/c.1096G>A patients were detected. The variant c.1066G>A (p.Ala356Thr) was detected in patient number 3. This variant has not been reported so far in patients with suspected deficiency of AAT1 and was not present in the population databases. The aforementioned variant affects the first nucleotide of exon 7 and alters the consensus sequence of recognition of the splicing acceptor of that exon. Bioinformatic splicing site calculation programs predict an alteration of the splicing site. Following the recommendations of the American College of Medical Genetics and Genomics [15], this variant must be classified as probably pathogenic. Due to the generation of a truncated protein, the most probable phenotype would be the “Null”.

**Figure 2:**
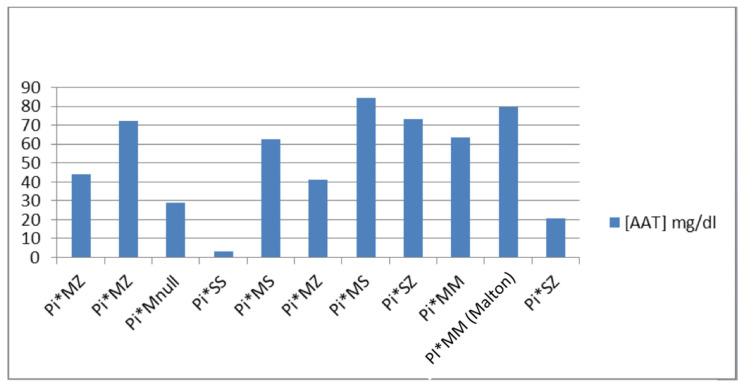
Presentation of the phenotypes in relation to the concentration of AAT1.

After the great proportionality between the band of alpha-1 globulins and the presence of a mutation in the *SERPINA1* gene, we predicted the phenotype of each patient according to the AAT1 concentrations and the available literature [6]. The phenotypes we found in the study were: (1) Pi*MZ: it has an average value of [AAT1] of 52.53 mg/dL, the maximum value being 72.1 and the minimum being 41.3; (2) Pi*MS: it has an average value of [AAT1] of 73.65 mg/dL, the maximum value being 84.6 and the minimum 62.7; (3) Pi*MM (Malton): only one case with an AAT1 concentration of 79.8 mg/dL is presented; (4) Pi*SS: only one case with a concentration of AAT1 <10 mg/dL is presented; (5) Pi*SZ: it has an average value of [AAT1] of 46.95 mg/dL, the maximum value being 73.4 and the minimum 20.5; and (6) Pi*MNull: only one case with an AAT1 concentration of 28.9 mg/dL is presented (Figure 2).

## 4. Discussion

In this study, we tried to verify if the SPE would be a useful tool as an opportunistic screening method for the deficiency of AAT1, and we found validity. Nevertheless, we want to make clear that it only represents an opportunity and not an alternative to diagnostic methods. From a public health point of view, the risk of severe consequences entailed due to the lack of diagnosis represents an incentive to standardize the screening of this disease. AAT1 deficiency is currently an entity diagnosed in the final stages of an evolved lung disease [16,17], and one which in those stages has a treatment that significantly decreases mortality [18] and slows the evolution of pulmonary emphysema [19]. In contrast, there are no data on how the disease would evolve by administering replacement therapy in patients who have not yet presented symptoms. With an early diagnosis, patients would benefit from the implementation of preventive measures and a targeted and specific action from an early time.

In any case, seeing our results, it is apparent that every patient who undergoes a SPE the band of alpha-1 globulins should be carefully evaluated. In addition, if an alteration is discovered, it will be necessary to determine AAT1. On the other hand, it should be imperative to take this entity into account in any patient with lung and/or liver problems. There are few bibliographic citations involving the usefulness of the SPE in the screening of AAT1 deficiency and they consider this method as not very sensitive and specific [20,21,22,23]. In Italy, prevalence studies have been carried out using different methods and creating controversy. In this way, Coude et al. [24] suggest that in high-risk areas adult population screening program, employing up-to-date genetic methods may be useful and additionally, Ottaviani [25], Ferrarotti [26], and, more recently, Scarlatta [27] illustrated that routine SPE is a suitable method for quantification of the alpha-1 globulin band and the detection of AAT1 deficiency.

In our study, we demonstrated its effectiveness with its high sensitivity (91%), allowing us to guide the rest of the tests—such as genetic ones—that should be limited due to their high cost. In other comparable studies [22], where similar values of AAT1 are taken as the cut-off point, the percentage of patients who subsequently presented mutations in the *SERPINA1* gene was very significant.

Regarding the impact of our study, it is important to consider that a decrease in the alpha-1 globulins of the SPE, confirmed with a lower than normal AAT1 concentration, is related to any type of mutation in the *SERPINA1* gene. If confirmed in larger population studies, it could be possible to elaborate a new diagnostic algorithm that can have a better and earlier approach in these patients. Taking the estimated figures of population with AAT1 deficiency into account, it could be deduced the great impact that this disease has not only in health but also within the economic resources since the current diagnosis based on the phenotyping of patients is three times more expensive than the method proposed in this work. According to the bibliography [6], the Null variants have an unknown relevance. The aforementioned variant detected by this study has not been previously described in the literature or in the population databases. It is a variant in a heterozygous state and the concentration for which it codes is significantly low (28.9 mg/dL). In this regard, we can affirm that certain variants are well studied while others are very far from their estimate. For all these reasons, one of the most relevant aspects of our study is the fact that when heterozygous variants appear, the estimation of mutations from the concentration of AAT1 is very complicated without either establishing ranges or being able to ensure which is the probable phenotype of the patients.

Concerning the limitations, we must mention the following: first, it was a cross-sectional study in which we performed an approach of the current population with a small sample size, although we must bear in mind that the AAT1 deficiency was included in the so-called “rare disorders”. In this sense, we aspire to carry out a future study with a greater representation of Aragon´s population (Spain). Secondly, we must remark that SPE will be useful in screening for AAT1 deficiency only if it is carried out in a basal state of health, since AAT1 is an acute phase reactant protein.

As final considerations, the AAT1 deficiency is a little-known entity that provides a useful model for the protein linkages, the effect of mutations, and therapeutic strategies for other conformational diseases. The number of patients who are diagnosed is minimal compared to official estimates and the fact that the substitute treatment gives good results marks the need to identify the patients who can benefit from it. Identifying patients, investigating the process, and offering a specific and targeted treatment and approach are the keys to the future of this disease. In this study, a solution to the first of the three points was proposed and it is foreseeable that the other two factors will progress successively to this. Considering the AAT1 deficiency as one of the most frequent life-threatening congenital diseases in adulthood, and taking into account that there are only a few patients diagnosed in Spain, it was demonstrated that our screening protocol was highly effective.

## 5. Conclusions

The serum protein electrophoresis is a useful tool for the opportunistic diagnosis of AAT1 deficiency. In all patients with alpha-1 globulins < 3%, the concentration of AAT1 should be quantified, since this cut-off point tends to anticipate a lower than normal concentration of AAT1 in baseline health conditions.

The relationship between a decrease in the concentration of AAT1 and the presence of mutations in the *SERPINA1* gene is close to 91%, but estimating the phenotype using the concentration of AAT1 is difficult, especially in heterozygous mutations.

## Figures and Tables

**Table 1:** Primers used for the sequencing of the *SERPINA1* gene.

EXON	Forward Primer	Reverse Primer
2	AAGGCTCCTTCCTGTCCAAG	CGCTGCTCTACATCCACTCA
3	CCATCAAGAGGGTGTTTGTGT	CGGATACCCACTCCACAAC
4	GTACTTGGCACAGGCTGGTT	ATGCATTGCCAAGGAGAGTT
5	GAGGGATGTGTGTCGTCAAG	TAGCAGTGACCCAGGGATGT
6	TAGTGTGGGTGGAGGACACA	CAGCCTGGGTCTTCATTTGT
7	GTGACAGGGAGGGAGAGGAT	CTGTTACCTGGAGCCCACAT

**Table 2:** General characteristics of the patients and relatives.

Patient	Age	Gender	α1%	Tobacco Exposure	Patient’s Symptoms	Symptoms in First-Degree Relatives
1	90	F	2.9	No	No	Sister and daughter with asthma
2	55	M	2.7	No	Psoriasis	No
3	36	M	2.8	Yes	Infertility	No
4	50	F	2.9	No	Infertility, spondyloarthropathy, and celiac disease	No
5	62	F	2.5	No	Liver fibrosis	Brother with liver insufficiency
6	83	M	2.6	No	No	Brother with lung emphysema
7	26	F	2.9	No	No	Maternal grandfather with lung emphysema
8	19	M	3	No	Liver fibrosis	Paternal grandfather with lung fibrosis
9	50	M	2.7	No	No	No
10	50	M	2.7	No	Diabetes, pancreatectomy	No
11	61	M	2.6	No	No	Two brothers with COPD

**Table 3:** AAT1 concentrations, distribution of mutations according to location in *SERPINA1* gene, and predicted phenotypes.

Patient	AAT1 (mg/dL)	Mutated Exon	Mutations	Phenotype
1	44.2	7	c.1096G>A (p.Glu366Ly) heterozygous	Pi*MZ
2	72.1	7	c.1096G>A (p.Glu366Ly) heterozygous	Pi*MZ
3	28.9	7	c.1066G>A (p.Ala356Thr) heterozygous	Pi*MNull
4	<10	5	c.863A>T (p.Glu288Val) homozygous	Pi*SS
5	62.7	5	c.863A>T (p.Glu288Val) heterozygous	Pi*MS
6	41.3	7	c.1096G>A (p.Glu366Ly) heterozygous	Pi*MZ
7	84.6	5	c.863A>T (p.Glu288Val) heterozygous	Pi*MS
8	73.4	5 and 7	c.863A>T (p.Glu288Val)/c.1096G>A (p.Glu366Ly)	Pi*SZ
9	63.6	Undiscovered	Undiscovered	
10	79.8	4	c.221-223delTCT (p.Phe76del)	Pi*MM (Malton)
11	20.5	5 and 7	c.863A>T (p.Glu288Val)/c.1096G>A (p.Glu366Ly)	Pi*SZ

## Data Availability

Not applicable.

## References

[1] Torres-Duran M., López-Campos J.L., Barrecheguren M., Miravitlles M., Martínez-Delgado B., Castillo S., Escribano A., Baloira A., Navarro-Garcia M.M., Pellicer D. (2018). Alpha-1 antitrypsin deficiency: Outstanding questions and future directions. Orphanet J. Rare Dis..

[2] Figueira Gonçalves J.M., Martínez Bugallo F., García-Talavera I., Rodríguez González J. (2017). Alpha1-Antitrypsin deficiency associated with null alleles. Arch. Bronconeumol..

[3] Irving J.A., Haq I., Dickens J.A., Faull S.V., Lomas D.A. (2014). Altered native stability is the dominant basis for susceptibility of α1-antitrypsin mutants to polymerization. Biochem. J..

[4] Lomas D., Parfrey H. (2004). Alpha 1-antitrypsin deficiency, Molecular Pathophysiology. Thorax.

[5] de Rienzo Modelo B., Chávez A.M.G., Guerra J.I.M., López N.G.J., Cedillo A.M., Ramírez R.I.L., Galdeano P.A., Altamira Y.M., García G.B., Contreras M.E. (2008). La importancia de la deficiencia de Alfa-1 antitripsina en el desarrollo de la enfermedad pulmonar obstructiva crónica y otras patologías pulmonares. Medigraphic Neumol. Cirugía Tórax.

[6] Vidal R., Blanco I., Casas F., Jardí R., Miravitlles M. (2006). Committee on the National Registry of Individuals with alpha-1 Antitrypsin Deficiency. Guidelines for the diagnosis and management of alpha-1 antitrypsin deficiency. Arch. Bronconeumol..

[7] American Thoracic Society/European respiratory Society Statement (2003). Standards for the diagnosis an management of individuals with alpha 1- antitrypsin deficiency. Am. J. Resp. Clin. Care Med..

[8] Snyder M.R., Katzmann J.A., Butz M.L., Wiley C., Yang P., Dawson D.B., Halling K.C., Highsmith W.E., Thibodeau S.N. (2006). Diagnosis of alpha-1-antitrypsin deficiency: An algorithm of quantification, genotyping, and phenotyping. Clin. Chem..

[9] Silverman E.K., Sandhaus R.A. (2009). Alpha1-Antitrypsin Deficiency. N. Engl. J. Med..

[10] Reilly J.J., Silverman E.K., Shapiro S.D. (2015). Chronic Obstructive Pulmonary Disease. Harrison´s Principles of Internal Medicine 2015.

[11] Jardi R., Rodríguez-Frias F., Casas F., Cotrina M., Vidal R., Miravitlles M., Pascual C. (1997). Molecular characterization of two variants of alpha-1-antitrypsin deficiency: Pi Mpalermo and Pi Plovel. Med. Clin. (Barc.).

[12] Jardi R., Rodríguez-Frias F., López-Talavera J.C., Miravitlles M., Cortina M., Costa X., Pascual C., Vidal R. (2000). Characterization of the new alpha-1-antitrypsin-deficient PI M-type allele, PI M (vall d’hebron) (Pro(369)-->Ser). Hum. Hered..

[13] Jardi R., Rodríguez F., Miravitlles M., Vidal R., Cotrina M., Quer J., Pascual C., Weidinger S. (1998). Identification and molecular characterization of the new alpha-1-antitrypsin deficient allele PI Y barcelona (Asp256-->Val and Pro391-->His). Mutations in brief no. 174. Online. Hum. Mutat..

[14] Lara B., Blanco I., Martínez M.T., Rodríguez E., Bustamante A., Casas F., Cadenas S., Hernández J.M., Lázaro L., Torres M. (2017). Spanish Registry of Patients with alpha-1 Antitrypsin Deficiency: Database Evaluation and Population Analysis. Arch. Bronconeumol..

[15] Richards S., Aziz N., Bale S., Bick D., Das S., Gastier-Foster J., Grody W.W., Hegde M., Lyon E., Spector E. (2015). Standards and guidelines for the interpretation of sequence variants: A joint consensus recommendation of the American College of Medical Genetics and Genomics and the Association for Molecular Pathology. Genet. Med..

[16] The Alpha-1 Antitrypsin Deficiency Registry Study Group (1998). Survival and FEV1 decline in individuals with severe deficiency of alpha-1 antitrypsin. Am. J. Respir. Crit. Care Med..

[17] Wencker M., Furhmann B., Banik N. (2001). For the Wissenschafliche Arbeitsgemeinschaft zur Therapie von Lungenerkrankunger. Longitudinal follow-up of patients with alpha-1-protease inhibitor deficiency before and during therapy with alpha-1-protease inhibitor. Chest.

[18] Stoller J.K., Fallat R., Schluchter M.D., O’Brien R.G., Connor J.T., Gross N., Kevin O., Robert S., Ronald C.G. (2003). Augmentation therapy with alpha-1 antitrypsin: Patterns of use and adverse events. Chest.

[19] Gotzsche P.C., Johansen H.K. (2016). Intravenous alpha-1 antitrypsin augmentation therapy for treating patients with alpha-1 antitrypsine deficiency and lung disease. Cochrane Database Syst. Rev..

[20] Miravitlles M., Jardi R., Rodríguez-Frías F., Torrella M., Pelegri D., Vidal R. (1998). Usefulness of the quantification of the alpha-1 serous protein band in the screening of alpha-1-antitrypsin deficiency. Arch. Bronconeumol..

[21] Jenkins M.A. (2000). Clinical applications of capillary electrophoresis. Status at the new millennium. Mol. Biotechnol..

[22] González-Sagrado M., López-Hernández N., Martín-Gil F.J., Tasende J., Bañuelos M.C., Fernández-García N., Arranz-Peña M.L. (2000). Alpha1-antitrypsin deficiencies masked by a clinical capillary electrophoresis system (CZE 2000). Clin. Biochem..

[23] Slev P.R., Williams B.G., Harville T.O., Ashwood E.R., Bornhorst J.A. (2008). Efficacy of the detection of the alpha1-antitrypsin “Z” deficiency variant by routine serum protein electrophoresis. Am. J. Clin. Pathol..

[24] Corda L., Medicina D., La Piana G.E., Bertella E., Moretti G., Bianchi L., Pinelli V., Savoldi G., Baiardi P., Facchetti F. (2011). Population Genetic Screening for alpha1-antitrypsin Deficiency in a High-Prevalence Area. Respiration.

[25] Ottaviani S., Trevisan M., Ferrarotti I., Gorrini M., Baldo R., Quargentan L., Luisetti M. (2013). Routine serum protein electrophoresis and detection of alpha1-antitrypsin deficiency. Eur. Respir. J..

[26] Ferrarotti I., Poplawska-Wisniewska B., Trevisan M.T., Koepke J., Dresel M., Koczulla R., Ottaviani S., Baldo R., Gorrini M., Sala G. (2015). How Can We Improve the Detection of Alpha1-Antitrypsin Deficiency?. PLoS ONE.

[27] Scarlata S., Santangelo S., Ferrarotti I., Corsico A.G., Ottaviani S., Finamore P., Fontana D., Miravitlles M., Incalzi R.A. (2020). Electrophoretic α1-globulin for screening of α1-antitrypsin deficient variants. Clin. Chem. Lab. Med..

